# Open Data In Neurophysiology: Advancements, Solutions & Challenges

**DOI:** 10.1523/ENEURO.0486-24.2025

**Published:** 2025-11-18

**Authors:** Colleen J. Gillon, Cody Baker, Ryan Ly, Edoardo Balzani, Bingni W. Brunton, Manuel Schottdorf, Satrajit Ghosh, Nima Dehghani

**Affiliations:** ^1^Department of Bioengineering, Imperial College London, London SW7 2AZ, United Kingdom; ^2^Dartmouth College, Hanover, New Hampshire 03755; ^3^CatalystNeuro, Benicia, California 94510; ^4^Scientific Data Division, Lawrence Berkeley National Laboratory, Berkeley, California 94720; ^5^Center for Computational Neuroscience, Flatiron Institute, New York, New York 10010; ^6^Department of Biology, University of Washington, Seattle, Washington 98195; ^7^Princeton Neuroscience Institute, Princeton University, Princeton, New Jersey 08540; ^8^McGovern Institute for Brain Research, MIT, Cambridge, Massachusetts 02139

**Keywords:** code, collaboration, datasets, neurophysiology, open science, sharing

## Abstract

Ongoing efforts over the last 50 years have made data and methods more reproducible and transparent across the life sciences. This openness has led to transformative insights and vastly accelerated scientific progress (Gražulis et al., 2012; Munafó et al., 2017). For example, structural biology (Bruno and Groom, 2014) and genomics (Benson et al., 2013; Porter and Hajibabaei, 2018) have undertaken systematic collection and publication of protein sequences and structures over the past half century. These data, in turn, have led to scientific breakthroughs that were unthinkable when data collection first began (Jumper et al., 2021). We believe that neuroscience is poised to follow the same path, and that principles of open data and open science will transform our understanding of the nervous system in ways that are impossible to predict at the moment. New social structures supporting an active and open scientific community are essential (Saunders, 2022) to facilitate and expand the still limited adoption of open science practices in our field (Schottdorf et al., 2024). Unified by shared values of openness, we set out to organize a symposium for open data in neurophysiology (ODIN) to strengthen our community and facilitate transformative open neuroscience research at large. In this report, we synthesize insights from this first ODIN event. We also lay out plans for how to grow this movement, document emerging conversations, and propose a path toward a better and more transparent science of tomorrow.

## Significance Statement

The adoption of open science practices is key to advancing research in neurophysiology. Drawing on insights from the inaugural Open Data in Neurophysiology (ODIN) symposium, this paper provides a much needed discussion on the current state of open science in the field, identifying current needs of the community and providing a roadmap. Detailing both obstacles and solutions to the wide adoption of open science practices, it constitutes an important resource for the neurophysiology community on the path to open science.

## Open Data in Neuroscience

Over the past half-century, many subfields of the life sciences have undergone a profound transformation through the adoption of open data and open science practices. These shifts have catalyzed scientific breakthroughs in fields such as structural biology and genomics, with systematic data sharing accelerating discoveries that were previously unimaginable. The availability of large-scale protein structure databases has led to advances in protein folding prediction (Jumper et al., 2021), while the widespread adoption of genomic repositories has revolutionized our understanding of genetic variation and disease (1000 Genomes Project Consortium, 2015). In contrast, neuroscience, and neurophysiology in particular, is still in the early stages of adopting open science values and practices.

Despite the vast amounts of neurophysiological data being generated, significant challenges remain in standardizing, sharing, and integrating these datasets across research groups. The potential benefits of open neuroscience are clear: improved reproducibility, broader collaboration, efficient data reuse, and deeper insights into the fundamental workings of the brain. Yet, barriers such as data heterogeneity, resource limitations, and institutional inertia continue to slow progress. Recognizing this gap, we organized the open data in neurophysiology (ODIN) symposium to bring together researchers, data scientists, and policymakers to discuss strategies for advancing open data practices in neurophysiology. For the full video versions of ODIN 2023 session recordings, see https://www.youtube.com/playlist?list=PLQVnU1OJzOn_mFlUL8aWQym4HfVvhlrGE. For summaries of each talk, see Appendix.

**Figure 1.**
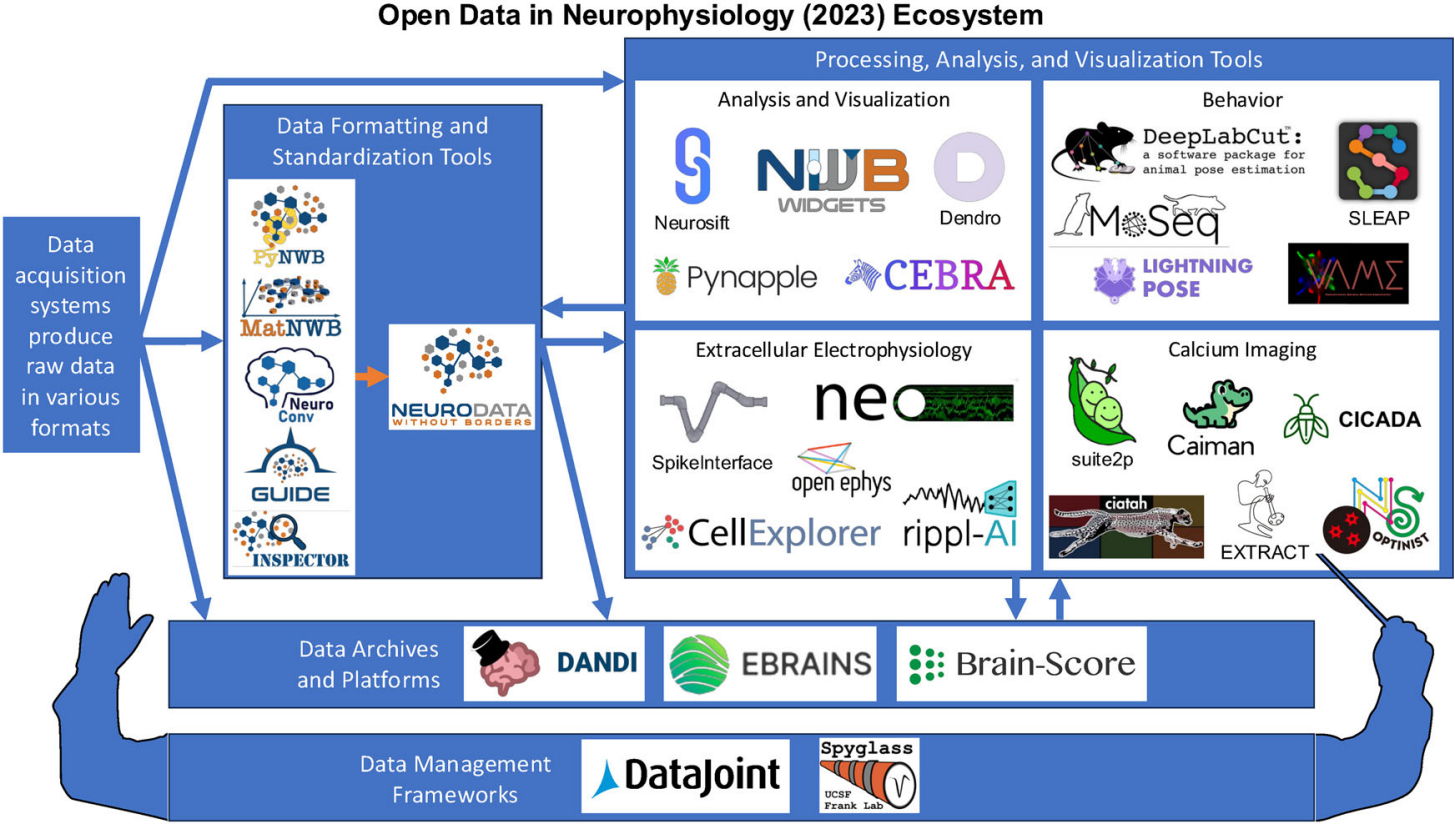
Snapshot of the current ecosystem of open-source neurophysiology tools. Note that the ecosystem is growing, and thus larger than what is depicted here. Included here are example tools that were presented or discussed during the ODIN 2023 symposium. See Table 1 for more information about each toolkit.

This paper synthesizes and expands upon key insights from the symposium. First, we explore the vast ecosystem of existing neurophysiology tools and resources (Fig. 1; Table 1). Specifically, we discuss devices, neuroinformatics, and platforms, followed by knowledge extraction, software, and modeling in neurophysiology. In each case, we present recent advances, as well as some of the major obstacles to progress in these domains. Next, we dive more deeply into current technical challenges in neuroinformatics that may be hindering widespread adoption of open science practices, also discussing potential solutions. Lastly, we provide a forward-looking perspective highlighting the current needs of the open science community, as well as recommendations for individuals and organizations seeking to engage with and promote open science practices. Throughout each of these sections, certain central themes consistently reemerge: (1) the need for robust infrastructure and standardized formats to enable seamless data sharing, (2) the cultural and institutional shifts required to foster openness and collaboration, and (3) the role of computational tools and machine learning in making large-scale neurophysiological data more accessible and interpretable. By identifying actionable steps and promising ongoing initiatives, we aim to provide a roadmap for researchers, institutions, and funding agencies seeking to contribute to a more transparent and collaborative scientific ecosystem.

**Table 1.** Neuroscience toolkits presented or discussed at ODIN 2023

Resource	Website	Tags
DANDI Archive	https://dandiarchive.org	data repository
EBRAINS	https://search.kg.ebrains.eu	dataset search, knowledge graph, web app
Brain-Score	https://www.brain-score.org	dataset search, benchmarks, model evaluation, web app
DataJoint	https://datajoint.com	data management, database, SQL, Python
Spyglass	https://github.com/LorenFrankLab/spyglass	data management, database, Python
Dendro	https://github.com/flatironinstitute/dendro	cloud computing, web app
Neurosift	https://neurosift.app	visualization, dataset exploration, web app
NWB GUIDE	https://github.com/NeurodataWithoutBorders/nwb-guide	data format conversion, desktop app
NeuroConv	https://github.com/catalystneuro/neuroconv	data format conversion, Python
Neo	https://github.com/NeuralEnsemble/python-neo	data format reading, Python
SpikeInterface	https://github.com/SpikeInterface/spikeinterface	spike sorting, electrophysiology, Python
rippl-AI	https://github.com/PridaLab/rippl-AI	SWR detection, electrophysiology, Python
OptiNiSt	https://github.com/oist/optinist	ROI segmentation, optical physiology, desktop app
Caiman	https://github.com/flatironinstitute/CaImAn	ROI segmentation, optical physiology, Python
EXTRACT	https://github.com/schnitzer-lab/EXTRACT-public	ROI segmentation, optical physiology, MATLAB
suite2p	https://github.com/MouseLand/suite2p	ROI segmentation, optical physiology, Python
DeepLabCut	https://github.com/DeepLabCut/DeepLabCut	pose estimation, behavior, Python
Lightning Pose	https://github.com/danbider/lightning-pose	pose estimation, behavior, Python
SLEAP	https://github.com/talmolab/sleap	pose estimation, behavior, Python
VAME	https://github.com/LINCellularNeuroscience/VAME	pose estimation, behavior, Python
MoSeq	https://github.com/dattalab/moseq2-app	video sequencing, behavior, Python
CEBRA	https://github.com/AdaptiveMotorControlLab/CEBRA	data analysis, latent space, behavior, Python
Pynapple	https://github.com/pynapple-org/pynapple	data analysis, Python

## Devices, Neuroinformatics, and Platforms

### New devices and high throughput acquisitions

Recent developments in neurotechnology have significantly improved the spatiotemporal resolution and throughput at which brain activity can be recorded. In turn, however, the resulting growth in available data has created significant challenges for data management, sharing and long-term storage.

#### Advances & examples

Advances in devices and acquisition systems are often the result of significant technical and engineering breakthroughs. For example, the development of multi-thousand channel electrocorticography (ECoG) grids now enables much denser and higher resolution mapping of brain activity than was possible using traditional clinical electrodes. This innovation was made possible by the development of smaller electrodes which can still yield a high signal-to-noise ratio. It was also critically facilitated by technical advances in thin-film microfabrication which allows electrodes to be more stably arranged along the brain’s curvilinear surface. The move toward wireless systems has also increased the efficiency of acute and chronic monitoring, while also reducing its intrusiveness (Tchoe et al., 2022). Similarly, the development of Neuropixels, a silicon probe which allows high-density simultaneous recording the activity of hundreds of neurons in awake and freely moving animals, has revolutionized the field of systems neuroscience, in which this technology is now ubiquitous (Jun et al., 2017). The latest version of the probe, Neuropixels NXT, aims to be more compact in design to increase the detail and scale at which electrophysiological neural activity can be recorded.

In optical imaging, the use of light sculpting and temporal multiplexing now enables cortex-wide, volumetric recording of neuronal activity across millions of neurons at single-cell resolution. Data yielded by this type of imaging technique will be critical to studying patterns of coordinated activity across distant regions of the brain (Demas et al., 2021). On a more fine-grained scale, combining voltage-sensitive fluorescent proteins activated by red light with blue-light-activated channelrhodopsins for neuronal stimulation now enables optical monitoring of electrical activity within neurons at high spatial and temporal resolution (Adam et al., 2019). Since this technique allows spikes and subthreshold voltages to be resolved, the data it yields could greatly enhance our understanding of within-neuron voltage dynamics, input-output relationships in local networks, and the plasticity rules that govern them.

Advances in tools like these facilitate the collection of vast amounts of high-quality neural data, and have the potential to yield transformative insights into the dynamics that govern the activity of entire neuronal populations.

#### Challenges & concerns

Arising directly from this significant improvement in resolution and throughput, however, is the challenge of managing very large datasets. With researchers increasingly collecting datasets comprising terabytes (TBs) of raw data, reliable large-scale data management systems are needed to store and disseminate them. If a researcher or laboratory opts to preserve all raw data collected, they must either devise their own data management system or rely on shared or externally maintained ones, such as institutional data management systems or large-scale online repositories like the distributed archives for neurophysiology data integration (DANDI) archive. Online data repositories in particular offer a very attractive solution. In addition to reducing the pressure for individual researchers to manage long-term data storage themselves, they are ideal for data dissemination. Of course, the challenge of data management remains, being simply displaced onto the managers of these online repositories. Comparisons with data management practices at institutions such as CERN—which handles 50–100 PB annually (European Organization for Nuclear Research, 2021)—suggest that current neurophysiological data repositories should be able to cope for now. However, if, over the next decades, the number of researchers around the globe opting to share their datasets publicly increases substantially, as we hope it will, these repositories will need substantial and reliable resources to scale at a rate that matches demand.

In addition to ensuring that repositories have the resources needed to scale their data management capacities, it is also important to consider carefully, as a community, what types of data should be preserved and what types of data can safely be discarded. Indeed, an alternative to storing TBs of raw data is to keep only a pre-processed, and thus much smaller, version of a dataset. Of course, this is not always appropriate, but there may be many cases where this approach provides an adequate compromise between data reusability and responsible use of resources. In such cases, a critical challenge for researchers is ensuring that the adopted compression and pre-processing steps do not discard information critical for future validation, reproduction of results or reanalysis of the data. For example, spike sorting methods must be carefully tuned to avoid yielding too many false positives or false negatives, complicating the extraction of reliable spike trains (Zhang et al., 2023). Thus, although pre-processed spiking data are much easier to share and reuse, critical information can easily be lost if the raw dataset they were extracted from is not also preserved. Guidelines for when and how to share pre-processed rather than raw datasets, or even for when to share protocols and analysis pipelines instead of datasets, are needed to ensure that storage resources are used responsibly, storage needs are met over the long-term, and the vital role of publicly available data in reanalysis and future discovery is protected (Dehghani, 2024).

A related concern is the trade-off between scale and interpretability. While recording from large populations of neurons can greatly increase the information contained in a dataset, this may not automatically lead to better scientific insights. As dataset sizes grow, so does the complexity of the data and often of the associated analyses. New analytical frameworks that move beyond traditional single-neuron analysis are needed to extract reliable network-level interpretations of brain activity. Collaborative efforts in this direction between experimentalists, theorists, data scientists, and engineers will be needed to ensure that neuroscience remains both data-rich and scientifically insightful.

In summary, high throughput neurophysiology offers unprecedented opportunities for understanding brain function. However, these advancements are accompanied by equally significant challenges. Only through a concerted effort that integrates advanced technologies with robust data management and scalable analytical strategies can the full potential of these innovations be realized.

### Neuroinformatic resources

A major goal of open science is to promote the sharing of datasets, protocols, and analysis tools across laboratories. Such practices reinforce transparency and scientific integrity, and can greatly increase research consistency and quality across laboratories. From an ethical point of view, measures that maximize data reuse also reduce the need for animal experimentation, thus directly contributing to animal welfare. However, a major impediment to resource sharing is the diversity of data formats, workflows and coding practices used by different groups. Common standards and guidelines are needed to manage the ever-growing volume and complexity of neurophysiology data discussed above. In response, the landscape of neuroinformatics tools is undergoing substantial transformations, driving an increased adoption of common data standards, the growth of standardized data repositories, and the development of interoperable workflow and data visualization tools.

#### Advances & examples

Ensuring that data collected and shared by one laboratory can easily be reused by a different group requires adopting and investing in common data standards. The neurodata without borders (NWB) ecosystem has emerged as a robust, multidisciplinary framework for organizing diverse datatypes—from neural activity recordings to experimental metadata—into a single, hierarchical format. Recent developments include support for cloud-based data access, as well as the integration of external resources and of a variety of application programming interfaces (APIs). These aim to increase usability and lower barriers to adoption for users from different backgrounds. In particular, more intuitive tools such as NeuroConv (https://github.com/catalystneuro/neuroconv) and NWB GUIDE (https://github.com/NeurodataWithoutBorders/nwb-guide) should simplify the process of converting proprietary data into the NWB format. Once standardized, these datasets can be stored and shared on repositories such as DANDI which, as of mid-2025, housed nearly a petabyte of data (DANDI, 2025). DANDI has also launched an associated JupyterHub platform to encourage cloud-based data analysis, further supporting the reusability of the data it hosts. This expansion of data standards and repositories hopefully signals a broad shift toward open science, where, in line with FAIR principles, data is *Findable, Accessible, Interoperable, and Reusable* (Wilkinson et al., 2016).

Complementing these advances in data standards and repositories are innovations in computational workflows and web-based visualization. With platforms like DataJoint, laboratories can recruit the assistance of external experts to create end-to-end computational workflows covering the entire lifecycle of their neuroscience projects—from data acquisition and animal management to spike sorting and behavioral analysis (Yatsenko et al., 2018, 2021). DataJoint resources can also be used to evaluate the operational maturity of existing workflows. Open-source solutions can then be identified to improve these workflows, for example by automating time-consuming steps using reliable artificial intelligence (AI)-based tools. Web-based tools are also changing how data can be analyzed and visualized. Open-source platforms such as Figurl (https://github.com/flatironinstitute/figurl), Neurosift (Magland et al., 2024), and Dendro (https://github.com/flatironinstitute/dendro-old) offer interactive, shareable visualizations that integrate seamlessly with data standards like NWB and repositories like DANDI which use these standards. These tools not only simplify data exploration, but also enhance collaboration as they enable scientists to share interactive figures and analyses through URLs. Altogether, automated workflows and containerization technologies show great promise in enhancing reliability, reproducibility and transparency in scientific research.

#### Challenges & concerns

Amid these promising developments, several challenges still remain. One challenge is ensuring that new technologies and resources can be easily adopted by different laboratories operating in very diverse research environments. Researchers establishing new laboratories should be encouraged to consult early with resource developers and incorporate standardized workflows from the beginning to streamline data management and analysis. For existing laboratories with well-established procedures, overcoming barriers to adoption will likely be much more difficult. In both cases, enhancing usability and providing robust user support remains critical. Importantly, standardizing commercially available data acquisition systems has the potential to greatly facilitate data sharing and analysis, removing most of the onus of data standardization from individual researchers. Notably, unifying these systems also presents potential advantages for clinical settings as it could streamline the management of data identifiability and privacy, thus reducing the risk of error and mismanagement.

By integrating standards like NWB, repositories such as DANDI, and innovative computational and visualization tools, the neuroinformatics community is making significant strides toward addressing the technical challenges of modern neuroscience research. Collaborative efforts between experimentalists, data scientists, and engineers are required to ensure that these new and developing tools can be productively adopted by the community.

### Platforms & collaborative initiatives

It is also increasingly recognized that improvements in collaborative platforms and research infrastructures are critical to addressing the reproducibility crisis and advancing our understanding of neurophysiology. Shared tools like integrated databases and common analytical frameworks are invaluable, openly accessible resources for the global scientific community. They can also bring together more distant research subfields, enabling, for example, cross-species neurophysiology comparisons. The current move toward inclusive, transparent, and collaborative research infrastructures is already accelerating discoveries and driving innovation in neuroscience.

#### Advances & examples

The Japan Marmoset Initiative, the OpenScope platform, the Allen Institute for Neural Dynamics, and the International Brain Laboratory (IBL) exemplify a shift toward large-scale, collaborative neuroscientific projects. The Japan Marmoset Initiative is a multi-institution initiative using data from genetically modified marmosets to advance brain mapping and disease modeling research. This initiative has yielded a detailed database of 3D structural, diffusion, and resting-state magnetic resonance imaging (MRI) data, as well as an in situ hybridization-based gene atlas. Projects using this data have the potential to yield significant insights, facilitating interspecies comparisons and enhancing our understanding of disease mechanisms such as those underlying Rett syndrome (Okano and Mitra, 2015; Hata et al., 2023; Skibbe et al., 2023).

A new platform inspired by astronomical observatories has also recently emerged. With the OpenScope platform (https://alleninstitute.org/division/neural-dynamics/openscope), the Allen Brain Observatory has made their high throughput pipelines for two-photon microscopy and Neuropixels recordings available for public use in order to democratize access to cutting-edge data collection. Researchers worldwide whose proposals are accepted receive neurophysiology data collected specifically for their project under highly standardized conditions. The double-blind review process for proposals, inspired by approaches to allocating time on shared telescopes, aims to ensure that resources are allocated based on scientific merit and equity considerations to foster both innovation and inclusivity (Koch et al., 2022; de Vries et al., 2023). Complementary tools like the OpenScope Databook have also been developed to help standardize and democratize data analysis and visualization techniques (https://alleninstitute.github.io/openscope_databook/intro.html). Efforts at the Allen Institute for Neural Dynamics have focused on both data management and computing for large-scale neuroscience. On the data management side, the institute aims to make data compliant with FAIR guidelines at acquisition, through robust metadata capture and early conversion to common data standards such as BIDS, NWB, and OME (Goldberg et al., 2005; Gorgolewski et al., 2016). They also aim to harness cloud computing infrastructures to reduce logistical hurdles related to moving and storing data, and to enable complete software and hardware environments to be shared via containerized pipelines. As these resources are publicly shared, these efforts will accelerate access to high-quality, standardized data and to fully reproducible workflows that can operate both in the cloud and locally.

Notably, these advances are directly aligned with the aims of the United States’ National Institute of Health (NIH)’s BRAIN (Brain Research through Advancing Innovative Neurotechnologies) Initiative, as outlined in the BRAIN 2025 report. Indeed, a core aim was to create a public, integrated ecosystem for seamless sharing of datasets and analysis tools. This ecosystem is currently distributed across a broad range of platforms which, collectively, house thousands of datasets (Tables 2 and 3). These platforms are poised to grow even further with the gradual introduction of mandates, like the BRAIN Data Sharing Policy, requiring researchers to share their data and code alongside their publications (https://braininitiative.nih.gov/vision/nih-brain-initiative-reports/brain-2025-scientific-vision). Together, the measures advanced by the BRAIN Initiative aim to ensure that research tools are shared broadly and that research data are made available to the wider community in a timely manner.

**Table 2.** BRAIN Initiative data archives

Archive	Link	Datatypes	Access restrictions
BIL (Brain Imaging Library)	https://www.brainimagelibrary.org	Confocal microscopy brain imaging	Some restricted datasets
bossDB (Block and Object Storage Service Database)	https://bossdb.org	Electron microscopy and x-ray microtomography	Public
DABI (Data Archive for the BRAIN Initiative)	https://dabi.loni.usc.edu	Invasive human neurophysiology	Some restricted datasets and requires registration
DANDI (Distributed Archives for Neurophysiology Data Integration)	https://www.dandiarchive.org	Cellular, systems, and behavioral neurophysiology	Public
NEMAR (Neuroelectromagnetic Data Archive and Tools Resource)	https://nemar.org	Electroencephalography (EEG) and magnetoencephalography (MEG)	Public
NeMO (Neuroscience Multi-Omic Data Archive)	https://nemoarchive.org	Multi-omics	Some restricted datasets
OpenNeuro	https://openneuro.org	Magnetic resonance imaging (MRI) and other types of neuroimaging	Public

These archives are generally public access, although some house restricted datasets. Most of these archives also allow embargoes, i.e., restricted access for a fixed period of time after initial publication.

**Table 3.** Generic archives that contain some neurophysiology data

Archive	Link	Datatype
Brain/MINDS Data Portal (Japan’s Brain Mapping Project)	https://dataportal.brainminds.jp	Includes marmoset structural and functional physiological data
Brain-Score	https://www.brain-score.org	Neurophysiology data
CRCNS (Collaborative Research in Computational Neuroscience)	https://crcns.org	Neurophysiology data
Dryad	https://datadryad.org	General research data
EBRAINS (European Brain ReseArch INfrastructureS)	https://ebrains.eu	Various types of neuroscience data
Figshare	https://figshare.com	General research data
G-NODE (German Neuroinformatics Node)	https://gin.g-node.org	Neurophysiology data
Zenodo	https://zenodo.org	General research data

All of these are public access.

The IBL has built a brain-wide map of neuronal activity during behavior by deploying standardized experimental protocols across 22 collaborating laboratories (https://www.internationalbrainlab.com). This initiative has generated an extensive dataset from nearly 33,000 neurons recorded using Neuropixels probes, offering an unprecedented opportunity to rigorously investigate the distributed processing of sensation, decision-making, action, and prior beliefs in a single task (International Brain Laboratory et al., 2021, 2023; Findling et al., 2023). Given the standardized, but distributed approach of the IBL, this dataset also provides an opportunity to better understand key issues of reproducibility in neurophysiology. Notably, analyses of the IBL dataset have shown that although standardized data collection can greatly reduce variability, even the use of different analytical methods and metrics on the same dataset can meaningfully alter the results of an experiment and their interpretation (International Brain Laboratory et al., 2024). Altogether, the IBL’s approach demonstrates the value of developing unified and sound methodologies covering the full lifecycle of an experiment, from data collection to analysis, and of harnessing open science practices to address complex neurophysiological questions and tackle the challenge of reproducibility.

Together, initiatives like these have the potential to considerably advance neurophysiology research. Given their scale, they are generally able to harness cutting-edge technologies and tackle complex operational and analytical challenges much more easily than individual laboratories can. Their commitment to open and reproducible research has also meant that the comprehensive, integrated databases and resources they have created are openly accessible to the global neurophysiology community. This offers critical opportunities for individual researchers worldwide, who may not have the requisite resources, data collection tools or software engineering capabilities, to contribute their expertise to the creation and analysis of complex datasets, and thus can help foster more inclusion and collaboration across the scientific community.

#### Challenges & concerns

The challenges faced by large-scale platforms overlap heavily with those faced by individual laboratories. However, given the specific mandate of several of these platforms to collect and disseminate vast datasets with high reuse potential for the broader research community, the consequences of not sufficiently addressing these challenges are arguably scaled up for these large platforms. Finding solutions that work for individual research groups, but can also scale to larger organizations, is therefore critical to ensuring that the deployment of large-scale platforms is effective, sustainable and constitutes an appropriate use of resources.

A key concern is again the issue of data management. As discussed in detail in the section on high throughput neuroscience, challenges include deciding what types of data, whether raw or processed, to preserve, determining how best to make datasets publicly available, and ensuring that the analytical frameworks being used are appropriate for the complexity and scale of the datasets. Another difficulty, also shared with individual laboratories, is the challenge of ensuring that the datasets collected by large-scale platforms include comprehensive metadata. Properly capturing, recording, and disseminating detailed metadata—including environmental conditions, experimental protocols, and subtle procedural nuances—is essential for contextualizing experimental outcomes and ensuring data can be appropriately reused, reproduced, and interpreted. Yet, tools for doing this easily and consistently are still lacking. Thus, for both large-scale and individual research efforts, there is a pressing need for user-friendly metadata collection and management tools. Lastly, an additional challenge, particularly faced by large-scale platforms handling human data, is ensuring that data privacy concerns, particularly in clinical settings, are properly addressed and that the deployment of open data practices does not compromise confidentiality.

To effectively address these challenges, experimentalists, data analysts, and software developers must collaborate on streamlining workflows in a way that is scalable, and preserves flexibility where necessary. Automated tools, when properly developed, can make the collection, processing and dissemination of data more standardized and robust, thereby reducing risks of data or metadata loss and better protecting data privacy. However, flexibility remains important as scientific needs can vary greatly between and even within experiments. Additionally, more centralized technical support platforms are needed at the intermediate level within institutions as resources for individual laboratories. Such core facilities can provide much needed expertise, assisting researchers in leveraging complex, emerging technologies without becoming overwhelmed by complex technical implementation details.

## Knowledge Extraction, Software, and Modeling

### Experimental models and data-driven approaches

Innovations in experimental design and analysis techniques also have the potential to significantly accelerate discovery and generate new knowledge in the field. However, to achieve this potential, these advances and the data they yield must be effectively disseminated to the community. We extend the discussion here into examples of new experimental models, as well as the collection and use of cutting-edge neurophysiology datasets, discussing how, through interdisciplinary efforts, these could be used to address long-standing questions in neuroscience about neural circuit development, the processing of complex sensory information, and the brain’s ability to generalize compared to AI systems.

#### Advances & examples

A recent innovation that has the potential to greatly improve our ability to study brain-like neural circuits using in vitro techniques is the development of cortical organoids. These three-dimensional structures capture key characteristics of real brains, like their diverse cell types and synaptic properties, which dissociated cultures typically cannot. Using optofluidic-CMOS multielectrode arrays and Neuropixels recordings, researchers have demonstrated the emergence of neuronal assemblies with sufficiently structured oscillatory dynamics to generate local field potentials (Sharf et al., 2022). This technology has the potential to be of great benefit to the community, opening up new avenues for exploring intrinsic cortical activity, network dynamics, and complex processes like learning and memory formation under well-controlled and replicable conditions.

In the study of sensory processing, closed-loop data-driven model training in which data is collected at the same time as neural networks are trained has emerged as a promising new technique. An example is the use of “inception loops” where stimuli presented to mice are modified in real time using deep learning in order to identify the stimulus patterns that most strongly excite specific neurons (Walker et al., 2019). Since the preferred stimulus patterns predicted for individual neurons are fed back into the experiment, the quality of the network’s predictions can be directly evaluated, thus avoiding some of the problems that arise when trying to interpret neuronal activity based on the outputs of black-box neural networks. Techniques like this are needed to move beyond overly simple interpretations of neuronal activity.

Large, high-quality multidimensional datasets also provide key opportunities to study complex neural processes and develop data-driven models. AJILE12 (annotated joints in long-term electrocorticography for 12 human participants) is an ECoG dataset collected in humans engaging in video-recorded and annotated natural behavior. It is openly available in NWB format and can be explored using a browser-based dashboard. When available to the broader neuroscience community, data exploration pipelines like these drive progress in a wide range of fields. For example, AJILE12 can be used for basic and clinical research, deepening our understanding of the relationship between neural activity and motor behaviors on the one hand, and enabling us to develop more robust brain–computer interfaces on the other (Peterson et al., 2021, 2022). Another highly reusable dataset is the MICrONS project dataset, which provides a unique opportunity for researchers to study the link between structural connectivity and functional output in the visual system (The MICrONS Consortium, 2025). Datasets like AJILE12 have also been integrated into educational initiatives like Neuromatch Academy (van Viegen et al., 2021) providing researchers in training the opportunity to cut their teeth on datasets and tools that are actually used in their field (https://github.com/neurovium/Neuromatch-AJILE12).

In addition to large-scale datasets, smaller-scale datasets collected by individual laboratories are critical to advancing research. The highly diverse research focuses of different groups ensure that together we are collecting a wide array of puzzle pieces. For example, the role of individual cell types in shaping circuit function in sensory cortices remains poorly understood, and across laboratories, the focus and methods of investigation often differ considerably. Research combining two-photon imaging with optogenetic control over specific cell types has helped illuminate how different inhibitory neuron populations shape aspects of auditory processing, like frequency discrimination and adaptation to temporal sound patterns (Natan et al., 2015; Tobin et al., 2025). If the infrastructure needed for laboratories to easily share and explore each other’s datasets and pipelines can be made available, the gaps between the puzzle pieces explored by different groups can be better bridged. This will pave the way for us as a community to obtain a much fuller picture of intricate brain functions, like sensory processing, than can be obtained from studying single experiments in isolation.

#### Challenges & concerns

Extracting new scientific knowledge from new experimental models and increasingly complex datasets also presents many challenges. In particular, it requires mathematical and computational frameworks that can handle the nonlinearity, non-stationarity, and high dimensionality inherent in neural data. Current models often fall short of capturing the full complexity of neural dynamics. Two primary avenues are available for addressing this problem. On the one hand, improved analytical models are needed to describe data in explicitly interpretable ways. On the other hand, numerical methods allow information to be more flexibly extracted from data, though this may be at the expense of rigorous interpretability. A combination of these approaches is likely needed to ensure that complex datasets are analyzed to their full potential.

The creation of digital twins of neural systems provides an interesting opportunity for improving analytical frameworks. A digital twin, in neuroscience, is an artificial system designed to faithfully mimic the dynamics of a specific neural system. In real brains, due to technical limitations, only a small portion of neural activity can be recorded at one time. In contrast, the behavior of a digital twin can be extensively and continuously monitored. Thus, a digital twin that has been well designed and trained to mimic a neural system, likely using machine learning, can generate a considerable amount of data that may, in turn, be ideal for constraining analytical models of the system under study. Certainly, for efforts like these to be successful, collaborations across fields of expertise are needed. Only then can we ensure that the rich datasets being collected are used to their full potential to constrain data-driven models and generate new insights about the brain.

### Neuroscience toolkits

Innovative neuroscience toolkits are being developed to ensure cutting-edge tools in neurophysiology research are readily accessible and usable for the broader community. Such toolkits now span the full spectrum of the data lifecycle, with applications to quantifying animal behavior, pre-processing electrophysiology data, interfacing with large-scale data infrastructures, and deploying advanced analysis algorithms, amongst many others. By increasing access to important technical tools, these toolkits help democratize science and facilitate collaborative efforts across the community.

#### Advances & examples

Pre-processing behavioral data to extract relevant information on pose and motion can be a very laborious process, if done manually. Sophisticated machine learning algorithms for motion capture and pose estimation have revolutionized this process, allowing users to automatically extract behavioral information from large data streams. Using DeepLabCut (https://github.com/DeepLabCut/DeepLabCut), for example, users need only manually label a small subset of their dataset to fine-tune the pre-trained pose estimation algorithm to their specific needs and the specific properties of their data. The fine-tuned algorithm can then be deployed on the entire dataset (Mathis et al., 2018). The automation of this process has resulted in a much greater availability of behaviorally labeled neural data. This, in turn, has paved the way for tools like CEBRA (https://github.com/AdaptiveMotorControlLab/CEBRA), which also uses deep learning, but to identify joint embeddings of behavioral and neural data (Schneider et al., 2023). For both DeepLabCut and CEBRA, operational resources have also been deployed to ensure that these tools are designed, shared and maintained for long-term adoption, community integration, and the democratization of scientific inquiry. Relatedly, motion sequencing (MoSeq; https://dattalab.github.io/moseq2-app) is an algorithm that automates the extraction of behavioral data from three-dimensional videos of freely behaving animals captured using depth cameras. By decomposing continuous behavior into distinct “syllables” and constructing behavioral state maps, MoSeq provides an unsupervised framework for understanding the sequential and repetitive structure of animal behavior (Wiltschko et al., 2020). This tool can be used, for example, to characterize how different external perturbations—from drug effects to environmental changes—affect behavior over time.

There is also a great need for precise and reproducible pre-processing and standardization of electrophysiological data. Accurate signal extraction is hindered by variable levels of background noise, differences in acquisition systems, high processing needs for large data volumes, and the complex relationship between intracellular neuronal excitability and extracellular signatures. Even differences in the data formats yielded by acquisition systems can make it difficult to ensure that key pre-processing steps like spike sorting are deployed consistently across experiments. SpikeInterface (https://github.com/SpikeInterface/spikeinterface) aims to address several of these problems by unifying diverse spike sorting algorithms and pre-processing routines into one Python package, enabling users to compare the outputs of different routines and use consensus metrics to identify reliable units and spikes. It allows for different computational backends, including cloud-based ones, and can be used for data compression to reduce file sizes and for creating web-based, shareable visualizations for quality control and manual curation (Buccino et al., 2020, 2023). Community engagement through active feedback is a core component of SpikeInterface’s development, ensuring that the tool continues to evolve to address current and new challenges in electrophysiology research.

Machine learning-based approaches, like those underlying DeepLabCut, CEBRA, and MoSeq, are critical to improving data processing, as they can better adapt to a dataset’s specific properties while also being efficiently deployed on large data volumes. Such algorithms have also been created for the detection of neural graphoelements, i.e., distinct waveforms or patterns observed in electroencephalography recordings. The detection of sharp wave ripples (SWR) in hippocampal recordings has traditionally been done using spectral methods which tend to generalize poorly across brain areas and neurological pathologies, and can bias the types of events that are detected. Machine learning techniques enable more flexible detection of SWRs, but are often tested only on a limited set of datasets. rippl-AI (https://github.com/PridaLab/rippl-AI) brings together in a single toolbox the five best performing algorithms developed in the context of an SWR-detection hackathon. Users can deploy all algorithms at once to obtain aggregated results, and also fine-tune them on their own data, as needed (Navas-Olive et al., 2022, 2024). This project demonstrates how community-based approaches to toolkit development can yield robust and widely applicable tools.

Notably, although the continued creation of new datasets, tools and models is of great value to the neuroscience research community, it can also lead to information overload. The European Brain ReseArch INfrastructureS (EBRAINS) Knowledge Graph (https://www.search.kg.ebrains.eu) is a large-scale data integration platform that emerged from the Human Brain Project and tackles this problem by uniting experimental data, computational models, and software tools within a semantic, linked data framework (Appukuttan et al., 2022; Bologna et al., 2022). In so doing, the initiative aims to enhance data discoverability and interoperability. EBRAINS also exemplifies a structural and cultural shift in research toward more collaborative and community-oriented science.

Together, these advances illustrate how long-standing technical challenges can be addressed by engaging members of the research community in developing cutting-edge tools, and investing in their deployment in user-friendly toolkits. This process critically democratizes access to state-of-the-art methodologies and creates a more interconnected, transparent, and sustainable research ecosystem.

#### Challenges & concerns

There are many challenges to ensuring that digital resources, like data processing pipelines and analysis tools, remain truly reusable over time. When a digital resource is first shared, its reusability can be maximized by ensuring it is well-documented and compatible with other widely used tools. Ensuring that it complies with relevant standardization practices can also greatly enhance its adoption by the research community. Meeting these expectations generally requires significant dedicated effort. In addition, preserving a digital resource’s scientific value over time requires continually maintaining and updating it, fostering community engagement, and providing reliable technical support. Open-source software is generally best maintained when processes are streamlined and automated, to the degree possible, and comprehensive testing is deployed. This is necessary, for example, to ensure that a digital resource produces reproducible results across different computational environments.

Meeting these standards requires time and technical expertise that laboratory members often do not have. Even if they do, transitions in project leadership—often due to the departure of PhD students or postdocs—can disrupt this process, and lead to an otherwise very useful resource becoming obsolete and unusable. The broader community of users, from experimentalists to computational researchers, must also be actively engaged to ensure that digital resources are refined and extended to new and relevant applications. However, facilitating community feedback and contributions can also be very time-consuming.

Overall, there are many challenges to ensuring the reusability of digital resources over the long-term. Broadly, they are likely best addressed through the hiring of dedicated software development and maintenance personnel. This, in turn, requires stable, ongoing funding. Adapting current research funding models to allow for such roles will be critical to ensuring that the digital resources produced by research groups continue to flourish.

### Modeling and benchmarking

On the modeling side of neuroscience, integrative approaches are also increasingly being embraced, as exemplified by toolkits like SpikeInterface and rippl-AI described above. Such approaches aim to bring together single, isolated models under a shared infrastructure, allowing them to be compared and even used in conjunction with one another. Successful integration requires the development of robust benchmarks against which existing and new models can be compared. This, in turn, requires high-quality, large-scale data covering a wide breadth of tasks and datasets to be available for model constraining and benchmarking. Overall, the task of moving toward more integrated computational neuroscience will require the community to bridge gaps between disparate datasets, metrics and models.

#### Advances & examples

Brain-Score (https://www.brain-score.org) is a platform for integrative benchmarking of models of the brain. By connecting large but previously disconnected datasets, Brain-Score creates a common ground for evaluating models on a wide range of neural and behavioral tasks, and identifying classes of models that best recapitulate the brain’s functions (Schrimpf et al., 2018, 2020). A framework like this not only provides robust constraints for model development and a mechanism for rapid model screening, but also offers an avenue for predicting experimental outcomes and optimizing data collection strategies (Bashivan et al., 2019; Schrimpf et al., 2024; Tuckute et al., 2024). Ensuring that biophysical and anatomical constraints are appropriately integrated into computational model design and training can also greatly improve their ability to capture real neural data. For example, traditional neural networks trained on detailed physiological data can learn to recapitulate flexible neural encoding of movement and to generalize across conditions, as the complexity of the data they are trained on requires them to identify robust solutions (Almani and Saxena, 2022; Saxena et al., 2022; Almani et al., 2024). This illustrates the importance of ensuring that high-quality data covering a wide breadth of task conditions is available for training and evaluating computational models.

New metrics and baselines are also needed to ensure computational models are robustly evaluated against real data. Research has shown that despite extensive remapping of place cells, animals can rapidly reapply learned responses across different environments. Manifold analyses deployed on this data show that task-specific representations can remain stable even through remapping, pointing to geometrical consistency across tasks (Wirtshafter et al., 2024). Such findings can provide concrete, and importantly non-trivial, targets and constraints for computational models. Separately, drawing on principles from shape theory, “shape metrics” enable high-dimensional neural representations to be compared across individuals or across models (Williams et al., 2021; Duong et al., 2023; Harvey et al., 2023; Pospisil et al., 2024). The variability measured can in turn be related to behavioral variability to determine how closely intertwined the two are in the specific brain region under study. Such metrics can also provide concrete targets for computational models, ensuring that they capture the patterns observed both within and across the brains of real subjects. Notably, these examples also highlight the value of using metrics derived from unsupervised analyses, in addition to supervised ones, to evaluate models.

Together, these efforts demonstrate the importance of developing integrated benchmarks evaluated on high-quality and varied datasets with robust metrics, and ensuring that benchmarking tools can be efficiently deployed on new models. Advances in this direction are critical for ensuring that computational research in neuroscience is well-integrated and grounded in real data.

#### Challenges & concerns

There are many challenges to integrating the field of computational neuroscience. First, many fields of research, such as the study of motor function, currently lack robust benchmarks against which models can be evaluated. Ideally, individual benchmarks should not be used as sole criteria for model selection. A wide range of benchmarks used in parallel can provide a more global and representative perspective on a model’s performance. In fields for which many different metrics and analysis techniques exist, like dimensionality reduction and manifold analysis, the wealth of options can also present a challenge. Researchers less familiar with the theoretical grounding of individual techniques may struggle to identify the approach most relevant to their dataset and research question. Well-documented toolkits that bring together a variety of related metrics and analysis techniques, and include clear usage guidance are needed to ensure that these tools are used correctly and in the appropriate setting.

Furthermore, benchmarks are only as robust as the data on which they rely. Thus, as mentioned above, high-quality and varied data is necessary for successful and reliable benchmarking. Thorough quality assurance procedures and rich metadata are indispensable to ensure that datasets used for benchmarking and for model training accurately reflect the neural activity and behaviors under study. Lastly, even once robust benchmarks are created, bringing together complex models built using different architectures and even software under a shared benchmarking platform, as Brain-Score has done for vision and language models, requires considerable time and effort. Community investment and sustained collaborations, as well as a willingness by the community to adopt standardized methodologies, are critical to the success of such projects.

## Key Technical Hurdles and Solutions

Having summarized many of the recent advancements in neurophysiology and justified their value to the field, we next examine in more detail specific challenges preventing widespread adoption of the technologies and practices. We also describe some possible solutions gleaned from other scientific areas that have experienced similar difficulties.

### Data standards

The United States’ government’s repository of Federal Enterprise Data Resources defines a “data standard” as a “technical specification that describes how data should be stored or exchanged for the consistent collection and interoperability of that data across different systems, sources, and users”. Such a specification comprises components like datatype, identifiers, vocabulary, schema, format, and API (https://resources.data.gov/standards/concepts/#data-standard). In this section, we discuss challenges to consistency and completeness when using data standards in neurophysiology.

#### Data standard strictness

How strictly or broadly the components of a data standard are defined greatly influences its applicability, usability and flexibility. For example, a very narrowly defined data standard might be easy to use, but applicable only to a handful of datasets. In contrast, the NWB standard aims to be applicable to the full range of neurophysiology datasets. Questions persist, however, about whether certain components, such as metadata vocabulary, and supported storage formats and APIs should be more narrowly specified to improve consistency and reusability. Existing perspectives generally favor providing recommended best practices and sensible defaults rather than imposing stringent rules, ensuring the NWB standard can adapt to the diverse needs found in neurophysiology research.

#### Ontologies

An ontology provides a structured framework for organizing and connecting descriptive terms, facilitating better understanding and communication within a field. Establishing links between descriptive terms used in neurophysiology and standardized ontologies can greatly improve clarity and communication. Associating a brain area studied in an experiment with its corresponding region in a widely used atlas is one example. Although resources like the National Center for Biotechnology Information Taxonomy, the Mouse Genome Informatics database, and the Neuroscience Information Framework Standard Ontology are available, they are often underutilized due to the additional effort required to consult them when entering identifiers into metadata. One proposed strategy is to develop interfaces that provide default options or infer metadata directly from the data, thereby simplifying and standardizing the metadata entry process. As above, balancing strict enforcement of accepted ontologies with the flexibility that keeps data usable remains an ongoing challenge.

#### Standardizing initial data recording

To promote the adoption of the data standards, data acquisition systems could be enabled to write raw data directly into standards like NWB. While this would greatly alleviate pressure on individual researchers and laboratories to handle data conversion, a direct conversion approach does present certain challenges. A first challenge concerns the comprehensiveness of metadata. Metadata includes information about data collection, experimental design, and subject details, all of which provide essential context for experimental data. However, many of these details are often collated after data acquisition begins from disparate sources like laboratory notebooks. As a result, the NWB files created during acquisition may initially lack compliance with the standard’s own metadata requirements. Extending the ecosystem of NWB-related tools to help researchers enter all available metadata at time of data collection, and add new information as it becomes available, could alleviate this problem. Metadata entry tools could also be used to encourage researchers to record a broader range of information, including environmental factors (e.g., temperature, humidity, and luminance), which is often omitted when recording experiment variables.

A second challenge is the problem of data stream alignment. It is very common in neurophysiology experiments for multiple time-based data streams, like neural and behavioral activity, to be collected in parallel, aligned to separate clocks. In fact, when using devices like the Neuropixels probe, data from individual probes, or even various channels within a probe, are not sampled exactly simultaneously, but are instead slightly offset from one another. The NWB standard requires data streams to be aligned to a common clock within a single NWB file. This alignment process is usually done offline after data collection, often using custom scripts, and can require user intervention to handle irregularities caused, for example, by hardware failures. The NWB standard could be modified to allow unaligned raw data streams to be recorded in their native clocks along with synchronizing pulse data. However, the wide variety in experimental setups and lack of universally accepted methods for sending synchronizing signals creates a considerable challenge for expanding data standards to accommodate idiosyncratically recorded raw data streams. Even if raw data streams could effectively be directly recorded in NWB files, it would be critical for data collectors to ensure aligned data streams are added to the files or to new files created from scratch before they are shared. Indeed, although some common alignment methods exist, such as SpikeGLX’s “tshift” method or SpikeInterface’s “phase_shift” method for synchronizing data from Neuropixels channels (Buccino et al., 2020; Karsh, 2024), many alignment protocols are laboratory specific. Sharing NWB files with only unaligned raw data streams would thus seriously impede the ability of end users to properly use the data, thus defeating one of the core goals of data standardization. Thus, although enabling data acquisition systems to write raw data directly into standards like NWB could be tremendously useful to the field, key obstacles remain to be addressed to create a sufficiently feasible and flexible solution.

#### Data curation

As mentioned above, experimental information like session or subject exclusions is frequently stored in laboratory notebooks or separate databases, yet integrating it into the data sharing process is vital for a complete understanding of dataset usage and proper interpretation of analyses. Since such annotations are often highly context-specific and free-form, determining how to embed them within data standards while maintaining sufficient flexibility for various experimental designs remains the object of ongoing discussions. These considerations again reflect the broader issue of standardizing experimental metadata in neurophysiology, where efforts must balance strict requirements with the fluidity needed to accommodate a spectrum of research approaches.

#### Provenance storage

Determining where to store provenance information—inputs, settings, and outputs from computational analyses—is another key consideration. Some standards and data management systems record provenance in separate files from the data. ALPACA, for instance, stores these data in Resource Description Framework (RDF) files (Köhler et al., 2024), while DataJoint uses a database-backed processing pipeline (Yatsenko et al., 2018). In contrast, data standards such as NWB aim to store all information related to an experiment within a single file. Provenance information could be integrated into the NWB standard by, for example, recording information similar to what is stored in RDF files directly into single NWB files. However, this would require adding another layer of complexity to creating NWB files and maintaining the NWB standard. Thus, leaving provenance management to external systems might be more practical.

### Common infrastructure and computational reproducibility

Neurophysiology data processing and analyses often depend on software packages with specific environmental and operating system requirements. Versions of these packages, their dependencies, and their installation environments are seldom recorded alongside analysis results. This omission complicates both the reproduction of results and the assessment of software bugs’ potential impact on data analysis outcomes.

#### Containerization as a solution

Containerization through tools like Docker presents a means of addressing reproducibility hurdles. A Docker container packages an application together with its dependencies, ensuring consistent behavior across different computing environments. Documenting this build process in a Dockerfile captures every command involved in creating the application’s environment and installing its required packages, facilitating the containerization process (Nüst et al., 2020). By sharing not only the data but also the entire computational setup used to process that data, researchers can substantially enhance the reproducibility of scientific findings. Further gains in computational reproducibility may also emerge from integrating Docker containers within data standards. The NWB standard includes an optional “source-script” field for storing a Uniform Resource Identifier linking to a container image and the scripts used for analysis, allowing other researchers to replicate a computational environment. This option could be expanded to allow individual data streams within a single file to be linked to distinct scripts or containers optionally operating on separate clocks, since NWB often holds multiple processed data streams produced by different recordings and analyses.

#### Harnessing AI

Integrating AI into neurophysiology research, particularly in the context of leveraging open data, comes with promise but also challenges. While exploring strategies for maximizing the benefits of these technologies, we must acknowledge the complexity, scale, and peculiarities of neurophysiology data. Several underlying considerations will shape the role of AI in neurophysiology research. One is determining whether the scientific community is ready to depend on large language models (LLMs) for data analysis given the opacity of their inner workings. A second is determining how doctoral researchers should balance time invested in developing computational expertise versus relying more on AI tools and specialists. A third is clarifying how much foundational knowledge is necessary to avoid misuse of AI techniques and misinterpretation of results by neuroscience researchers with non-computational backgrounds.

As the community moves to adopt more AI methodologies, three primary barriers nonetheless hinder their broad application to neurophysiology data:

- **Experiment Diversity and Dataset Quality.** The heterogeneity of experimental designs makes it difficult to generate consistent data for meta-analyses, highlighting the need for “AI-ready datasets”. The challenge is that these datasets must be exceptionally large in sample size, well-organized, and mutually consistent in order to support the training and validation of effective AI models (Deng, 2012; Chen et al., 2021).
- **Common Vocabulary for Neural Patterns.** The lack of consensus on a common vocabulary to describe neural patterns (such as ripples, bursts, avalanches, etc.) impedes the generalizability of AI methods. A shared terminology is vital for effective communication throughout the community. Shared terminologies have been more widely adopted, for example, for describing the classes of neural computations based on underlying low-dimensional manifolds (Vyas et al., 2020), but are still lacking or incomplete when it comes to describing dendritic, neuronal, or microcircuit activity. As a result, although automated methods, such as sequential spectral density approaches or deep neural networks, have been proposed for spindle detection (Dehghani et al., 2011; Kaulen et al., 2022), SWR analysis (Ramirez-Villegas et al., 2015; Liu et al., 2022; Navas-Olive et al., 2022), and cell-type classification based on spiking waveforms (Barthó et al., 2004; Peyrache et al., 2012; Teleńczuk et al., 2017), their applicability and reach remain limited due to a lack of universal agreement on conventions.
- **Annotations.** It is currently uncommon for neurophysiology datasets to receive annotation beyond the original experimenter(s). By inviting external users to contribute annotations to datasets across all archives, unexpected meta-patterns may be discovered and overall “AI-readiness” increased (Raddick et al., 2009; Banerji et al., 2010). Common standards such as BAABL (Ly et al., 2024) and hierarchical event descriptors (Bigdely-Shamlo et al., 2016) could also be used to standardize and improve data annotations for even greater reusability.

#### Data quality and benchmarks

Relatedly, it is important for the community to be able to assess the usability of a dataset prior to training an AI model. Reliable metrics are needed to evaluate and transparently report the quality of both raw and processed data sources. These can provide helpful feedback for improving future experiments. The MRI Quality Control tool provides a compelling example (Esteban et al., 2017). Using such metrics as benchmarks, public leaderboards for model training can be hosted to incentivize competitive groups to achieve top ranks (Schrimpf et al., 2018, 2020), thus accelerating progress in research. Such strategies point to a strong need for community-driven tools and collaborative approaches, which will become increasingly important as AI plays a larger role in neurophysiology research. Further developments in each of these areas are expected to improve the quality of open data over time.

#### Community engagement and collaboration

Finally, several obstacles exist when it comes to engaging the neurophysiology community in using new tools, as well as in collaborating on projects. First, despite the potential of new tools like containerization and AI methodologies, widespread adoption requires broader user familiarity and more accessible infrastructure for the neuroscience community. Researchers must also be supported in managing the costs associated with long-term archiving and maintaining of outputs like container images and trained neural networks. Addressing these obstacles is vital to ensuring the viability of improved reproducibility and analysis methods for the broader neurophysiology community. Second, many laboratories have and continue to develop their own frameworks and terminologies tailored to specific experimental protocols. These lab-specific rules and approaches can lead to confusion and limit collaboration. While this diversity offers certain advantages, establishing a clearer common ground and shared tools would facilitate communication, reduce misunderstandings, and support more consistent use of effective methodologies.

## Looking Ahead

In this section, we discuss recommendations for strengthening the open science community moving forward. Specifically, we will discuss the importance of building community and of appropriately harnessing LLMs as a new and potentially revolutionary tool. We also list current community needs, as well as recommendations for both the practicing neuroscientist and the neuroscience science community as a whole.

### Building communities

#### Building a community

Open science thrives in a well-supported ecosystem where community-based governance and communication can flourish (Saunders, 2022). The nascent but growing ODIN community will require robust mechanisms for dialog and self-regulation, ideally emerging organically from within the community itself. A prime example of this model is Wikipedia, which thrives under self-imposed rules and a transparent decision-making process. Unlike transient tools like team communication platforms, a wiki provides a durable, public, and cumulative resource for community discourse (Kelder et al., 2012). Engaging in quality discussions and integrating these alongside the data itself will ensure accessibility and transparency for the wider public.

#### Regular meetings

The enthusiasm shared during this symposium suggests a strong desire for it to continue on a regular basis. These meetings are envisioned as key catalysts for fostering a robust ODIN community, and drawing together diverse voices from across the neurophysiology and systems neuroscience spectrum. By maintaining open communication channels and featuring varied perspectives, we hope to enrich our collective knowledge. In addition, we hope that continuing to share these talks on widely used and open video sharing platforms will ensure broad, public accessibility and engagement.

### Harnessing LLMs

The advent of advanced LLMs such as *OntoGPT* (Caufield et al., 2023) and *BrainGPT* (Luo et al., 2024) heralds a transformative shift in how scientific information can be processed, understood, and utilized. These models have demonstrated a remarkable ability to distill and predict complex patterns from vast datasets, suggesting a potential role in enhancing user interaction with neuroscientific databases. AmadeusGPT showcases an innovative application in this direction, using LLMs to convert natural language descriptions of animal behaviors into executable analysis code, thereby facilitating interactive behavioral research, and increasing its accessibility (Ye et al., 2023). Tools like these exemplify the potential of LLMs to help bridge gaps between complex biological knowledge and expertise in computational analysis, enhancing scientists’ ability to access and analyze neuroscience data.

LLMs also have the potential to help researchers engage more effectively with existing scientific knowledge, for example through enhanced literature searches and dynamic knowledge base augmentation via scientific journal content distillation. For example, BrainGPT has been specifically trained to anticipate the outcomes of neuroscience experiments by ingesting extensive portions of the neuroscientific literature. Its proficiency, as demonstrated by the BrainBench benchmark, was shown to surpass that of human experts in distinguishing between true experiment results and modified abstracts (Luo et al., 2024). This suggests a potential use for LLMs in inductive reasoning (Wang et al., 2023) where an LLM trained on open data is harnessed for hypothesis generation and experiment planning. Capabilities like these also point to a future where LLMs could be used to reliably navigate and summarize existing scientific knowledge. It is important to note, however, that LLMs are subject to hallucinations. For this reason, BrainGPT is not currently enabled to perform this type of task (Huang et al., 2023), and this potential use remains a matter of conjecture for the moment. However, such models could be used to refine literature search mechanisms, enabling researchers to rapidly locate relevant studies and datasets. By processing queries using LLMs trained on the latest research and reviews, search engines could offer more contextually aware search results, reducing the time spent on literature reviews and increasing the relevance of the information retrieved.

OntoGPT’s approach to enhancing knowledge bases through natural language processing highlights another very promising application for LLMs (Caufield et al., 2023). By helping construct and refine knowledge bases, LLMs can facilitate more accurate and dynamic querying of complex data structures. When it comes to managing extensive open neuroscientific data repositories, integrating LLMs could dramatically improve the precision and scope of data retrieval processes, enabling researchers to generate interconnected insights from disparate datasets. They could also be used to ensure that data curation and query management follow standard nomenclature and are consistent with open neuroscience practices. For example, LLMs can assist in linking new data entries with existing ontologies and suggest updates to improve the comprehensiveness and utility of a database.

Thus, as LLMs evolve, we should be able to leverage them not only to manage and query existing data, but also to anticipate and prepare for future research developments. Continuous updates to LLM training sets and algorithms and fine-tuning, using methods like LoRA (Hu et al., 2021) and retrieval augmented generation (Lewis et al., 2021) techniques, will be essential to maintaining their effectiveness and relevance to the neuroscientific context. Overall, incorporating LLMs into open data in neuroscience promises to be very useful, as such a dynamic, forward-looking tool could not only serve current user needs, but also adapt to and anticipate future scientific challenges.

### Community needs

For open science to advance in neuroscience, it is critical to understand and address the needs of the community. Table 4 summarizes key community needs identified during the symposium. Most broadly, these include a need for improved resources and tools, including educational ones, that make open science practices easier to adopt for users of all backgrounds. However, another key need is for changes to funding and incentives that ensure users can invest the time and effort required to adopt open science practices. In particular, incentives that recognize and reward data sharing, collaborative model development, and interdisciplinary efforts are needed to build a truly open and innovative neuroscience research community.

**Table 4.** Community needs and actions for advancing open science

Category	Actions	Key concepts
Guidance	• Provide community guidance on sharing methodologies, datatypes (raw, processed).	Provenance, shared methodologies, standardized metadata, unified ontologies
	• Standardize required and recommended metadata types.	
	• Select and unify ontologies for metadata standardization.	
	• Define essential provenance information for shared data.	
Tool development	• Enhance tools for data compression, conversion, sharing, and analysis.	Cloud solutions, data compression, data pooling, metadata entry, tool benchmarking
	• Develop cloud-based data access and analysis solutions.	
	• Establish benchmarking platforms for model and theory evaluation.	
	• Develop platforms for tool comparison.	
	• Support large-scale data pooling and annotation.	
	• Simplify metadata entry through user-friendly interfaces.	
	• Improve automated metadata capture tools.	
	• Enable on-the-fly data annotation of anomalies during experiments.	
	• Improve ability to detect and filter anomalous data.	
Research	• Improve models for understanding complex data.	Advanced model zoo, automated data labeling, data quality metrics, model benchmarks
	• Create benchmarks and metrics for model evaluation.	
	• Develop data quality assurance metrics.	
	• Innovate on automated data labeling for enhanced data reuse.	
Databases	• Maintain centralized databases for datasets, methodologies, and tools.	Centralized databases
	• Facilitate community feedback mechanisms for shared resources.	
Knowledge & education	• Create knowledge graphs for describing entities and their relationships, and for linking disparate databases.	Knowledge graphs, online resources, training workshops
	• Continue to develop online resources and training for data processing and analysis tools.	
Funding & incentives	• Support community engagement and multi-laboratory collaborations.	Community engagement, core facilities, multi-laboratory collaboration, open-source support
	• Fund technical personnel for open-source software maintenance.	
	• Fund the creation of core facilities in research institutions that provide centralized technical expertise to individual laboratories.	
	• Encourage and facilitate adoption of new technologies and open science practices.	
	• Invest in scaling data storage solutions.	

### Recommendations for the practicing neuroscientist

As we continue to innovate and advocate for community-wide advancements in open science, practicing neuroscientists have several opportunities to engage with the existing open science practices. This section makes recommendations that span the entire lifespan of a project and can greatly improve the reproducibility and efficiency of one’s research. Depending on their projects and access to resources, individual research groups may find certain recommendations more relevant, helpful or feasible to implement than others. We recommend identifying these priorities and approaching the adoption of open science practices incrementally.

#### Data management and sharing plan

**Figure 2.**
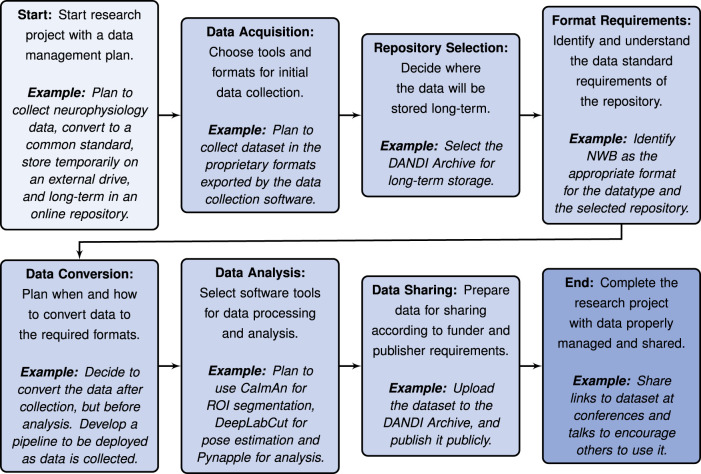
Data management plan flowchart, with example.

It is important to prepare a data management and sharing plan early in the research process. Funders like the NIH and many scientific journals now require open sharing of data collected under their grants and for publication, respectively. Deciding early on which repository to use, understanding its data standard requirements, and planning the workflow from data acquisition to publication can greatly facilitate the process. Adopting standards such as NWB early in data acquisition can also streamline the process and save time by ensuring consistency is maintained through data processing, analysis, and publication. See Figure 2 for steps of a recommended workflow. Additional recommendations are listed below for optimizing the process of selecting and converting to a data standard:

1. Identify early on the **repository** where your data will be deposited. Understanding the **data standard requirements** of the chosen repository and whether it needs to conform to specific standards such as NWB or BIDS will critically shape future steps.
2. Consider how you will **manage, process, analyze, and visualize** your data. The software tools you will use may have limitations on the data formats they accept, and the formats they can output.
3. Plan to **convert data** to a common standard **as early as possible** in the data lifecycle: from acquisition through processing, analysis, and up to publication and sharing. Early adoption streamlines workflows, avoids the need to refactor custom code down the road, and enhances the reusability of data, saving time and resources.
4. **Automate**, to the extent possible, the process of **converting your data** into the required standard using tools like NWB GUIDE. If conversion is done at the acquisition step, make sure that the proper metadata is included. If certain post-processing steps are required routines in your laboratory, make sure to track the details of how they were run and include the relevant information during data conversion to standardized formats.

#### Documentation and metadata

**Figure 3.**
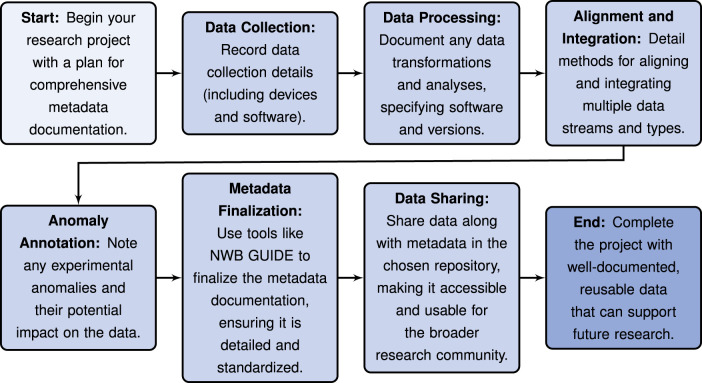
Documentation and metadata flowchart.

1. Provide **thorough and structured metadata** to enable effective reuse of your data and use by researchers who are not familiar with your project. As AI and machine learning methods become increasingly integrated into neurophysiology data analysis, this will also ensure your datasets are **AI-ready** with rich metadata, greatly enhancing their reusability and the reliability of subsequent findings. See Figure 3 for steps of a recommended workflow when storing and sharing data. Specific recommendations about the types of data to include are expanded on below:
  - Document the **source-script** and any other processes used to generate the dataset, even if they are not mandatory fields in your chosen data standard.
  - Include comprehensive details about the **devices**, **software versions**, and **analysis algorithms** used during the experiments.
  - Record any **stimuli** presented during the experiments, and include a detailed table specifying which stimuli were presented when.
  - Clearly describe how **neural, behavioral and stimulus data streams** were aligned temporally.
  - Record key subject descriptors, like **genotype**, referencing external databases for standard definitions where applicable.
  - Annotate any **anomalies or unusual occurrences** during data collection that might affect subsequent analyses.
2. Utilize tools like **NWB GUIDE** for user-friendly and automated capture of important metadata, minimizing effort and enhancing standardization.

#### Contributing to existing datasets

When running robust and well-validated analyses on existing datasets, consider sharing a derivative dataset comprising the results of these analyses. Contributing, for example, the results of dimensionality reduction analyses applied to a high-dimensional neural dataset or of pose estimation applied to behavioral recordings can greatly enhance the effective value of an existing dataset. For example, if the data is already shared on DANDI, addition of derivative to the existent dandiset or publishing it as a stand-alone dandiset is fairly simple and straightforward.

#### Utilizing existing tools

**Figure 4.**
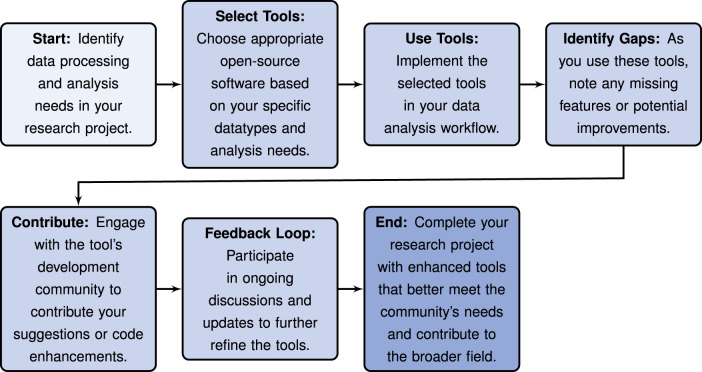
Tooling flowchart.

Several steps can help optimize your use of existing tools. See Figure 4 for an example workflow. Additional recommendations are provided below:

1. **Choosing Open-Source Software:** Due to the complexity of neurophysiological data analysis, it is advisable to use established open-source software packages, where possible and appropriate. These are less prone to errors and are continually vetted by the community. Examples include:
  - **Spike Sorting and Processing:** Consider tools like SpikeInterface and KiloSort.
  - **Calcium Imaging Data Processing:** Consider tools like suite2p and Caiman.
  - **Pose Estimation:** Consider tools such as DeepLabCut and SLEAP.
2. **Contributing to Tool Development:** If existing tools lack certain features or could be improved, contribute your enhancements back to the project. This type of collaboration:
  - Allows the community to verify the robustness of the new feature.
  - Enhances tool functionality and utility for the entire community.
  - Accelerates scientific discovery and increases the robustness of research outcomes.
  - Builds a culture of reuse and improvement, aligning with open science principles.

#### Developing new tools

If you develop new tools from scratch, it can be very valuable to share these with the broader community. To maximize the robustness, usability, and findability of these tools, it is particularly helpful to:

- **Share** the code on a platform like GitHub that enables robust version-control, as well as user feedback and contributions, ideally under a license that is highly permissive for code reuse and adaptation.
- **Document** the code by including at minimum a README explaining the tool’s intended use, the programming language it is designed in, its dependencies, usage examples, and ideally an interactive tutorial users can run in the cloud. More detailed recommendations can be found in previously published articles (Eglen et al., 2017).
- Make a **plan** for long-term maintenance and promotion of the tool. This may require investing financial resources and hiring of dedicated personnel, but is generally critical for the longevity and usability of an open-source tool.

### Community-wide recommendations

For major advances in open science practices to take hold in the field of neurophysiology, broad structural changes are needed that will improve the support and incentives offered to individual practicing neuroscientists. These changes will need to be implemented and adopted by the structures that oversee funding and resource allocation (i.e., funding agencies, funding committees, and universities), publication (i.e., journals, conferences, and their editorial boards), and academic career advancement (i.e., universities, departments, and tenure committees).

#### Allocation of funding and resources

The adoption and implementation of open science practices can be very time-consuming. It is therefore critical that laboratories have access to funding that is available or earmarked for hiring staff or students whose primary task is setting up and managing the laboratory’s open science pipelines, tools, and datasets. It is also critical to ensure that laboratories have access to enough funding to maintain the equipment and tools required to create, deploy, and maintain their open science pipelines, tools, and datasets.

At a broader scale, since many different laboratories in a same department, university, or region require similar open science resources, opportunities must be created for groups to secure stable and ongoing funding for the creation and maintenance of shared resources, also known as core facilities. Such resources could include long-term data storage facilities, high-performance computing clusters, and in-house research software engineering, data management, and data analysis support services. Access to internal or external expert consultants is a particularly under-considered need. Indeed, as discussed in an article of *The Transmitter* (Voigts, 2023), the breadth and depth of skill required to run high-quality neuroscience studies has grown considerably over the last decades, to a level that is likely unreachable for most individual neurophysiology laboratories. To empower research laboratories to maintain a high quality of research, it is critical that researchers come together to identify the resources they need access to in order to best deploy their skills and allocate their time. Funding opportunities must therefore be readily available to address these resource needs. The creation of such shared resources would not only greatly alleviate pressure, reducing the breadth and depth of technical expertise required of individual laboratories, but could also greatly reduce resource waste by improving research quality across the board.

#### Recognition and enforcement in publications

It is important that we ensure that the value of open science contributions such as new datasets and tools is properly recognized in journals and conferences. To this end, clear submission and evaluation criteria must be developed enabling reviewers to fairly evaluate open science contributions for key features such as expected short-term and long-term usability and usefulness to the community. Developing effective criteria and directing reviewers to attend to these will not only improve recognition of these contributions to the field, but also draw wider attention to them.

Alongside improved recognition, mandatory adoption of open science practices must be more broadly enforced. In the long-term, a clear goal for the field is that all papers be published with their accompanying datasets and code made as public as ethics and privacy considerations allow. These datasets and codebases should be made available in formats that allow them to be easily deployed to confirm and extend the published results. Of course, these requirements cannot be enforced overnight as laboratories must be given the time and resources to build up their ability to meet them. Journals should nonetheless continue to progressively increase their adoption and enforcement of such policies. Early enforcement measures include directing reviewers to formally consider data and code sharing as part of their assessment of the submission quality. Actual data and code review could also begin to be included in the review process. To avoid overburdening reviewers, however, this would require authors to make their data and code very easy to access and deploy. Eventually, as open science practices become much easier to adopt, authors should be allowed to withhold data or code only when absolutely necessary, for example for ethical and privacy reasons.

Enforcement of open science practices will also need to come from the funding side of research. Major funding agencies have already taken measures requiring researchers to include a feasible data management plan when applying for funding (Government of Canada, 2025; European Research Council, 2025; National Institute of Health, 2025). With these policies being comparatively new, however, it is not clear how carefully these plans are reviewed for feasibility and compliance, and to what extent and in what manner data sharing is enforced. As data sharing becomes the norm in the field, it will be important to establish clear and streamlined criteria for evaluating data management plans and for monitoring compliance with these plans. It may also be necessary to adopt penalties, like funding restrictions, for failure to comply with a data sharing plan without proper justification. Lastly, funding agencies should strongly consider extending these policies to code and software which are arguably often easier to share, and critical to fully understanding and assessing research results.

#### Recognition in research career evaluations

Finally, contributions to open science by members of the academic community, including undergraduate students, graduate students, postdoctoral fellows and professors, should be officially recognized as substantive contributions. Such contributions include, but are not limited to, standardizing and sharing datasets, sharing well-organized and documented codebases, and sharing and maintaining software tools, The impact of these shared resources can in turn be measured, for example, through citation counts, rates of reuse by the community, and the resource’s role in advancing the field. Current efforts by the NIH to develop, through a community challenge, a data sharing index (the S-Index) represent an encouraging step in this direction (Freelancer, 2024), and will hopefully be accompanied by better recognition of other important open science contributions like software and codebase sharing. Data and code publishing platforms should also enable users to mint unique identifiers like digital object identifiers, streamlining the process by which contributions are cited and disseminated. Members of the academic community should also be encouraged to highlight their open science work in their CVs, university applications, and funding applications. Admissions committees, departments, hiring and tenure committees, and funding and grant review committees should in turn be directed to give these contributions proper credit in the evaluation of candidates’ academic records.

## Concluding Remarks

This first ODIN symposium highlighted a growing momentum in neurophysiology research to incorporate the values and practices of open science. The key takeaways from the symposium are summarized in Table 5. Innovations revolutionizing the quality and quantity of data we collect have been complemented by the development of robust data standardization and sharing platforms, along with a variety of computational resources for data processing, analysis and visualization. Large-scale data collection efforts are tackling the challenges of reproducibility and reliability in the field, with centralized approaches providing access to high-quality data collection pipelines and decentralized ones encouraging collaborative protocol and analysis designs. However, significant challenges remain, particularly for laboratories with limited resources, where incorporating open science practices can be daunting and time-consuming. Furthermore, when laboratories do invest in adopting these practices, the time and effort required are often not sufficiently recognized by the traditional incentive structures of academic research.

**Table 5.** Key takeaways for advancing open science in neurophysiology

Category	Description
Education and training	Education and training of neuroscientists at every level is crucial for ensuring open data practices are effectively adopted and utilized.
Funding investment	Funding investment in the development, dissemination, and maintenance of open-source tools and infrastructure is necessary to support long-term sustainability and reliability of research outputs. Sponsors that value open data must be prepared to fund the health of the ecosystem, which includes supporting practicing neuroscientists, tool disseminators, and continuous maintenance/development to keep tools up to date.
Research methodologies	Improving research methodologies by establishing benchmarks and standardized methodologies for data analysis and model evaluation will improve the reproducibility and comparability of research findings. Appropriately harnessing large language models (LLMs) and AI tools to enhance data analysis, literature search, and hypothesis generation could also significantly improve research quality and pertinence.
Tools	Development of robust and user-friendly tools for data management, analysis, and sharing is essential to support the adoption of open science practices across laboratories with varying resources. Enhancing metadata quality and standardization is critical for the reusability and reproducibility of shared datasets, and comprehensive searchable metadata will greatly improving data utility. Such practices have the potential to help address important ethical considerations, like animal use in neurophysiology research, as optimal reuse of existing datasets can help keep animal use to a minimum.
Career paths	Alternative career paths should be established within academia to support individuals skilled in data management and analysis, whose work is less focused on specific research hypotheses. Such positions would provide much needed job opportunities and security, while helping bridge the gap between traditional academic hierarchies and the increasingly complex technical landscape of neuroscience research.
Culture and social infrastructure	Progressive changes in culture and social infrastructure are necessary and must occur alongside changes in incentives and credit assignment. Tool development and dataset contributions should receive greater appreciation and formal recognition (e.g., from hiring and promotion committees). Participating in this transformation is essential for fostering an environment that values and rewards open science.

Funding sponsors, publishers, and institutions wield the power to drive collaborative progress and sustain momentum. Their active support and recognition of the time and effort invested by researchers in open science initiatives are crucial for enabling this pivotal change in modern neuroscience. By acknowledging the value of open science practices, they elevate the entire field. Through incentivizing open data practices, funding robust infrastructure, and promoting tool dissemination, they create an environment where open science becomes a central pillar of neurophysiology research.

Meanwhile, we encourage researchers to actively engage in open science practices and leverage existing resources. By participating in the communities that build and use advanced tools, individuals can discover solutions to challenges they face, and tap into valuable community support. Where solutions are lacking, researchers can provide feedback reflecting their specific research needs, increasing the likelihood that future iterations will address those needs. Thus, while transformative impact arises from collective action with the much needed support of funders and institutions, it is essential to recognize the power of individual voices in shaping this action.

Overall, we anticipate gradual, collaborative progress in the field, rather than an overnight transformation, engaging researchers, sponsors and institutions. We advocate for acknowledging and celebrating symbiotic developments, which together will propel us toward more open, transparent, and impactful science. In this context, the ODIN symposium (intended as a bi-annual event) can serve as a vital platform for sustaining momentum, sharing novel developments, and addressing the evolving needs of the community.

## Synthesis

Reviewing Editor: Michaël Zugaro, CNRS, Collège de France, Inserm Decisions are customarily a result of the Reviewing Editor and the peer reviewers coming together and discussing their recommendations until a consensus is reached. When revisions are invited, a fact-based synthesis statement explaining their decision and outlining what is needed to prepare a revision will be listed below. The following reviewer(s) agreed to reveal their identity: Joshua Siegle, Anne Churchland.

This manuscript provides a thorough overview of the the inaugural Open Data in Neuroscience (ODIN) symposium (MIT, 2023). This synopsis of the current landscape and future directions will be of interest to all practicing neuroscientists who must grapple with the challenges involved in sharing their own data. This is an important topic as the field continues to generate and analyze large-scale datasets, which demand innovative approaches for effective management, sharing, and utilization. The authors highlight contributions to open science, such as data standards (e.g. NWB), platforms (e.g. DANDI), and collaborative projects like MICrONS and OpenScope. The paper concludes with forward-looking recommendations to promote transparent and reproducible science.

Strengths

- The paper addresses a relevant and timely topic, emphasizing the importance of open data in enhancing reproducibility and accelerating scientific science discovery in neuroscience.

- The manuscript covers a wide range of topics, from neurophysiological tools to collaborative projects and future directions, offering a wide survey of the field. This breadth is valuable to readers, as it introduces them to tools, platforms, and concepts that they may not have considered before.

- The 4 Tables - Neuroscience toolkits presented or discussed at ODIN 2023, BRAIN Initiative data archives, Generic archives that contain some neurophysiology data, and community needs and actions for advancing open science - summarize the key formats/tools, allowing the reader to easily access and identify useful resources tailored to their research needs while highlighting the various resources available to the community.

- The inclusion of concrete steps and 'Recommendations for the Practicing Neuroscientist' is a strength of the manuscript. By outlining practical measures - such as adopting standardized formats, maintaining detailed documentation, and leveraging evaluated tools - it promotes researchers to implement open science practices effectively.

Major concerns

- One overall issue with this manuscript is that readers may come away feeling that the proposed solutions are not commensurate with the scale of the problems at hand. Symposium attendees called for changes that will require both big financial outlays and serious social engineering. For example, addressing a "need for a paradigm shift in how neuroscientific data is handled" or "maintaining a low barrier to entry [for using] sophisticated tools" are laudable but challenging goals. Neither will happen without major updates to the incentive structure of neuroscience. However, most of the concrete advice given by the authors centers around changes that can be made by individual researchers, with only generic recommendations provided for "funding sponsors, publishers, and institutions." As long as there is no penalty associated with making data available upon reasonable request, and tenure committees do not weight data sharing efforts as heavily as publications in prestige journals, it seems like it will be hard to effect meaningful change in the data sharing space.

- Regarding the form, the manuscript could benefit from much tighter organization. The breadth of topics covered sometimes detracts from a focused narrative, making it challenging for readers to discern key takeaways. I would consider restructuring the manuscript to highlight a few central themes with supporting examples to improve clarity and focus. A more integrative narrative could better emphasize overarching themes and their implications for advancing open science, making it easier for readers to engage with the content and apply it to their work. Currently, the manuscript comes across as a point-by-point summary of the conference.

- The content about the details of the conference organization (e.g., the fact that it was similar to COSYNE , had succinct presentations and included a Q&A after talks) didn't seem very relevant to the major goals of the paper. The paper will have a bigger impact if it reads less like a conference proceeding and more like a technical report or a perspective piece. This would be helped both by the organizational changes described above, and also by smaller changes. For instance, replacing "A highlight of the talk was the detailed overview of the growing NWB ecosystem" with "A highlight of this efforts is the growing NWB ecosystem."

- The manuscript lists some of the important tools for processing physiology data, such as Caiman and Suite2p, but omits some important ones: Kilosort (for spike sorting of electrophysiology data), YASS (a spike sorter from the Paninski lab, see biorxiv 2020) and DREDge (A method for motion correction in large scale recordings from Neuropixels; see Windolf et al, Biorxiv 2023).

Minor Concerns

- The visual presentation of Figure 1 is informative but could be enhanced by organizing the categories for flow. To also include an overhead title for NWB, EBRAINS, Brain-Score, DANDI.

- Some sections are verbose and could be edited for clarity without losing overall message.

- The pipelines, such as Figure 2 for Data Management, might benefit from additional detail/visualization, to also include the tools/platforms, to understand when these tools should be implemented.

- Line 337 should also reference the International Brain Laboratory's paper on reproducibility in electrophysiology datasets (https://elifesciences.org/reviewed-preprints/100840v1). This paper uncovered that variability in electrode targeting led to failures of reproducibility, as did the lack of standardized inclusion metrics (such as RIGOR- Recording Inclusion Guidelines for Optimizing Reproducibility). This might be a more specific example of the importance of standardization compared to the example the authors stated (line 340 states that "different analytical methods used on the same data could nonetheless yield distinct interpretation" which is a bit vague).

Others

- OpenScope section: wherever "Brain Observatory" is mentioned, it should read "Allen Brain Observatory" instead.

- Line 1051: "An" → "A"
